# Concentration of Testosterone Glucuronide in Urine from Women with Breast Tumours

**Published:** 1977-12

**Authors:** Renzo Grattarola


*Br. J. Cancer* (1977) **36**, 818

### *Letters to the Editor*

### Concentration of Testosterone Glucuronide in Urine from Women with Breast Tumours. M. K. Jones, I. D. Ramsay and W. P. Collins *Br. J. Cancer* (1977) **35**, 885.

Sir,—In a high proportion of women with benign breast disease the androgenic activity is abnormal and the premenstrual endometrium hyperplastic (R. Grattarola, *Senologia* (1977) **2**, 19). As the result of this study I suggested that abnormal androgenicity might be the hormonal condition for converting a benign to a malignant breast disease; so I am not surprised when the authors of this paper claim that there is no difference in the concentration of the testosterone glucuronide from patients with benign or malignant breast disease. What surprises me is their conclusion; that their results, which come from two groups of women with either benign or malignant tumours of the breast, do not concur with my results, which showed that in women with breast cancer and hyperplastic endometrial pattern, the amount of testosterone excreted is above that of women whose breast was free from any disease, as ascertained by clinical and mammographic examinations, and whose endometrial pattern was normal.

The comparison made in this study is clearly quite different from mine and consequently the conclusions must be different too.

3 October 1977

Renzo Grattarola
Director, Hormone Research Laboratory,
Istituto Nazionale per lo Studio e la Cura
dei Tumori, via Venezian. 1, Milan.


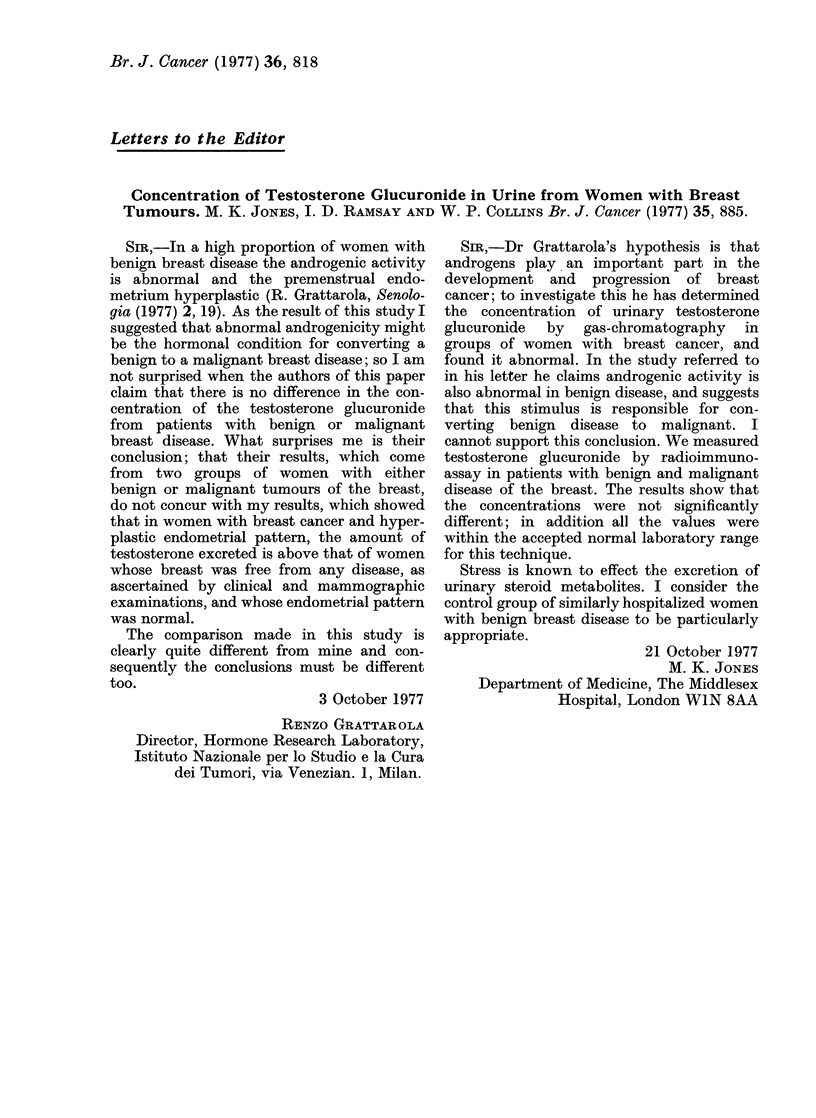


## References

[OCR_00009] Jones M. K., Ramsay I. D., Collins W. P. (1977). Concentration of testosterone glucuronide in urine from women with breast tumours.. Br J Cancer.

